# Escin suppresses HMGB1‐induced overexpression of aquaporin‐1 and increased permeability in endothelial cells

**DOI:** 10.1002/2211-5463.12622

**Published:** 2019-04-10

**Authors:** Changjun Chen, Songgang Wang, Jiying Chen, Xiaolin Liu, Mengchen Zhang, Xi Wang, Weihua Xu, Yayun Zhang, Hao Li, Xin Pan, Meng Si

**Affiliations:** ^1^ Department of Orthopedics Qilu Hospital of Shandong University Jinan China; ^2^ The Key Laboratory of Cardiovascular Remodeling and Function Research Chinese Ministry of Education Chinese Ministry of Health the State Shandong Province Joint Key Laboratory of Translational Cardiovascular Medicine Qilu Hospital of Shandong University Jinan Shandong China

**Keywords:** aquaporin‐1, barrier dysfunction, endothelial cells, escin, high mobility group protein 1

## Abstract

Escin, a natural triterpene saponin mixture obtained from the horse chestnut tree (*Aesculus hippocastanum*), has been used for the treatment of chronic venous insufficiency (CVI), hemorrhoids, and edema. However, it is unclear how escin protects against endothelial barrier dysfunction induced by pro‐inflammatory high mobility group protein 1 (HMGB1). Here, we report that escin can suppress (a) HMGB1‐induced overexpression of the aquaporin‐1 (AQP1) water channel in endothelial cells and (b) HMGB1‐induced increases in endothelial cell permeability. This is the first report that escin inhibits AQP1 and alleviates barrier dysfunction in HMGB1‐induced inflammatory response.

**Abbreviations**
AQP1: aquaporin‐1
AQP: aquaporin
CVI: chronic venous insufficiency
DAPI: 4′,6‐diamidino‐2‐phenylindole
EC: endothelial cell
HMGB1: high mobility group protein 1
HUVEC: human umbilical vein endothelial cell
MTT: 3‐(4,5‐dimethylthiazol‐2‐yl)‐2,5‐diphenyltetrazolium bromide

High mobility group protein 1 (HMGB1), a nuclear factor and a secreted protein involved in the stabilization of nucleosomes, is an important pro‐inflammatory mediator, usually derived from activated monocytes and macrophages or passively released by necrotic or damaged cells 1. An increasing number of studies have demonstrated that HMGB1 plays an important role in pathological processes of endothelial permeability, which can increase paracellular gap formation accompanied by loss of peripheral organized actin fibers, dissociation of cell–cell junctional cadherins, and development of central stress fibers, a phenotypic change associated with increased contractile activity, indicating increased barrier dysfunction and hyperpermeability of endothelial cells (ECs) 2, 3. On the other hand, it is reported that a severe infection might lead to the death of ECs or putatively to apoptosis, though whether HMGB1 could induce EC apoptosis remains to be elucidated, resulting in microvascular barrier dysfunction and albumin hyperpermeability, in other words, leakage of protein‐rich edema fluid into organs 4, 5. What is worse, capillary leakage could lead to increased tissue edema (the swelling of tissues due to excessive fluid accumulation), cell hypoxia and the excessive local accumulation of inflammatory cytokines, which directly lead to tissue and cell damage and even contribute to the development of multi‐organ failure 6. Hence, it is important to find a therapy to reverse the HMGB1‐induced permeability‐increasing effect to ameliorate hyperpermeability of ECs and even edema.

Escin, a natural triterpene saponin mixture obtained from *Aesculus hippocastanum* (Hippocastanaceae), the horse chestnut tree, has been used medically for the treatment of chronic venous insufficiency (CVI), hemorrhoids, and edema resulting from cerebral ischemic damage, trauma, or operation 7. With its clear‐cut anti‐edematous, anti‐inflammatory and venotonic properties and excellent tolerability in the clinic, escin has benefited millions of patients suffering from CVI, hemorrhoids and peripheral odema 7. Previous literature has shown that escin combined with glucocorticoids is effective in reducing the volume of edema and exudate in models of inflammation during the initial exudative phase, indicating that escin has a significant anti‐inflammatory effect during the acute stage 8, 9. Recently, Domanski *et al*. 10 reported that escin can potently induce cholesterol synthesis rapidly followed by a marked fall in actin cytoskeleton integrity in ECs, resulting in reduced migration, alleviated endothelial monolayer permeability, and inhibition of nuclear factor‐κB signal transduction. However, knowledge of how escin exerts its protection from endothelial barrier dysfunction induced by pro‐inflammatory HMGB1 is still limited. Thus, the mechanism of the anti‐edematous effect of escin could be more than inhibition of inflammation, and changed water permeability of human ECs might be involved in this process. Therefore, in this study, we examined the potent effects of escin on HMGB1‐induced aquaporin (AQP) 1 changes in ECs.

Aquaporins are water channel proteins, a highly conserved family of water‐specific, membrane‐channel proteins expressed in many epithelial and endothelial cell types that facilitate rapid transcellular and transepithelial fluid transport. They are involved in a wide range of physiological functions and human diseases 11. AQP1, the best characterized of the AQP family, is specifically and strongly expressed in most microvasculature ECs outside of the central nervous system, such as in kidney, brain, and eye, and functions in osmotically driven water transport 11. Moreover, it has been reported that AQP1 might facilitate EC migration and possibly be involved in tumor angiogenesis, whose mechanism is probably related to membrane protrusion (lamellipodium) formation at the leading edge of migrating cells resulting from actin cleavage and ion uptake 12. It has been reported that AQP1 may aid the absorption of excess water from the interstitial space into the capillaries 13. AQP1 inhibitors may thus have aquaretic and antiangiogenic activity. Hence, due to the numerous important roles of AQP1 in physiology, selective inhibitors of AQP1 may provide novel treatment opportunities for a variety of human disorders, such as glaucoma, brain edema, and even certain types of tumor growth 14. However, only a very few potent and chemically attractive blockers, with limitations in usefulness because of toxicity or lack of specificity, have been reported to date 15, 16. Therefore, the discovery of potent and selective inhibitors is highly desirable for future research, and an evaluation of natural medicinal plants with limited toxicity as sources of active compounds would help in this.

Based on these considerations, the current study was designed to answer several questions, including whether HMGB1 increases the expression of AQP1 and whether escin could inhibit increased expression of AQP1 induced by HMGB1. Then we investigated morphological change of filamentous actin (F‐actin) during HMGB1 stimulation and the potential effect of escin on this in cultured ECs. We also aimed to detect a possible effect of HMGB1 and escin on the permeability of monolayer ECs. Hence, we designed the study to broaden the panel of AQP modulatory agents and clarify the potential roles of escin in HMGB1‐induced hyperpermeability and even in edema and inflammation.

## Materials and methods

### Cell culture

Primary human umbilical vein endothelial cells (HUVECs) were donated from Key Laboratory of Cardiovascular Remolding and Function Research of Shandong University (Jinan, Shandong, China). Briefly, the cells were cultured in 5% CO_2_ at 37 °C in Endothelial Cell Medium supplemented with 5% fetal bovine serum, 1% endothelial cell growth supplement, and 1% penicillin/streptomycin solution (ScienCell, San Diego, CA, USA). The cell medium was refreshed every 36 h. Trypsin (0.25%; Solarbio, Beijing, China) was used for digestion and the cells were passaged for 1–2 min every 3–4 days.

### Cell viability

Metabolic activity can be evaluated by measuring the activity of the mitochondrial enzyme succinate dehydrogenase using the 3‐(4,5‐dimethylthiazol‐2‐yl)‐2,5‐diphenyltetrazolium bromide (MTT test). This test is widely used *in vitro* to evaluate the toxicity of plant extracts. We applied the MTT test to evaluate the potential toxic effects of escin on HUVECs. Cells were exposed to an increasing concentration (10, 20, or 30 μg·mL^−1^) of escin in the presence of HMGB1 (100 ng·mL^−1^) or incubated with HMGB1 (100 ng·mL^−1^) alone for 24 h. The cells were incubated with 20 μL MTT and placed in a 37 °C incubator for 4 h. Then, the cell culture suspension was removed and 100 μL of dimethylsulfoxide was added to the microplate to dissolve the formazan substance. Absorbance of the solution at 490 nm was measured by a microplate reader.

### Immunocytochemistry

ECs were cultured in 12‐well plates containing a poly‐lysine‐treated glass slide in each well and treated with HMGB1 (100 ng·mL^−1^, Novoprotein, Shanghai, China) or HMGB1 (100 ng·mL^−1^) combined with escin (used at 10, 20, or 30 μg·mL^−1^; Shanghai yuanye Bio‐Technology Co., Ltd, Shanghai, China) or control, and then fixed with 4% paraformaldehyde for 15 min. The coverglasses, on which the ECs grew, were removed and rinsed with PBS (Solarbio) at room temperature for 20 min. After being washed three times with PBS, cells were blocked with 5% BSA (Guangzhou Saiguo Biotech Co., Ltd, Guangzhou, China) in PBS for 30 min at room temperature, and subsequently incubated with primary antibody against AQP1 (1 : 200, Proteintech Group, Inc., Rosemont, IL, USA) at 4 °C overnight. Then ECs were washed twice with PBS (pH 7.4) before being exposed to FITC‐labeled goat anti‐rabbit IgG secondary antibody (1 : 200; EarthOx, Millbrae, CA, USA) at room temperature in the dark for 30 min. Nuclei were stained with 4′,6‐diamidino‐2‐phenylindole (DAPI; Solarbio) for 1 min. Images were captured using a fluorescence microscope (Olympus DP72, Tokyo, Japan) at fixed exposure time.

### Western blot analysis

To determine the expression of AQP1, we carried out western blot analysis. ECs were washed with ice‐cold PBS, and then lysed with RIPA (Epizyme, Shanghai, China) lysis buffer containing PMSF (1 : 100; Hangzhou Fude Biological Technology Co., Ltd, Hangzhou, China) for 30 min on ice and then centrifuged at 12 000 ***g*** for 15 min. The protein concentration was determined using the bicinchoninic acid protein assay kit (Beyotime Biotechnology, Shanghai, China) according to the manufacturer's manual. The lysates were heated in 95 °C for 10 min in protein sample loading buffer (Epizyme) and then the samples were kept at −20 °C until further analysis. Protein (10 μL) was separated on 12.5% SDS/PAGE (Epizyme) and transferred to a poly(vinylidene difluoride) membrane (Epizyme). The membrane was blocked with 5% skim milk in Tris‐buffered saline (TBS) containing 0.1% Tween‐20 [TBST; 50 mm Tris/HCl (pH 7.4), 150 mm NaCl and 0.1% Tween‐20] for 1 h at room temperature. The membrane was then incubated with primary antibodies, including polyclonal rabbit anti‐human AQP1 (1 : 600, Proteintech Group, Inc.) and monoclonal mouse anti‐human actin (1 : 800; Yeasen, Shanghai, China) at 4 °C overnight. After an intensive wash, the membrane was incubated with horseradish peroxidase‐conjugated secondary antibodies (1 : 500; EarthOx) at room temperature for 1 h. After being washed three times with TBST, the bands were visualized using an Omni‐ECL Femto Light Chemiluminescence Kit (Epizyme) and quantified using GE Amersham Imager 600 (General Electric Company, Bosto, MA, USA), and the expression levels were normalized to β‐actin. Each experiment was repeated at least three times.

### F‐actin cytoskeleton staining

ECs were grown on glass coverslips and then treated with HMGB1 (100 ng·mL^−1^) or HMGB1 (100 ng·mL^−1^) combined with escin (10, 20, or 30 μg·mL^−1^) or control for 24 h. After stimulation, the cells were fixed in 4% formaldehyde (Solarbio) at 4 °C for 15 min and then permeabilized with 0.2% Triton X‐100 (Solarbio) for 5 min and blocked in 5% BSA in PBS, prior to being incubated with TRITC‐phalloidin (1 : 200; Yeasen) at room temperature in the dark for 1 h. Finally, nuclei were stained with DAPI. Images were collected using a confocal laser scanning microscope (LSM710; Carl Zeiss, Jena, Germany) at fixed exposure time.

### Endothelial monolayer permeability

EC monolayer permeability was assessed by measuring the rate of transfer of FITC‐labeled dextran across the monolayer over time. The ECs (1 × 10^5^ cells) were grown on 4 μm pore Transwell filters (Costar®; Corning Inc., Corning, NY, USA) until confluent. Then the monolayers were treated with control or HMGB1 (100 ng·mL^−1^) or HMGB1 (100 ng·mL^−1^) combined with escin (10, 20, or 30 μg·mL^−1^) at 37 °C for 24 h. FITC‐labeled dextran (10 mg·mL^−1^) suspended in 100 μL PBS was applied apically after exposure, 10 μL of medium was aspirated from the lower chamber 90 min later, and FITC‐labeled dextran was measured with a fluorescence spectrometer (LS‐50B; PerkinElmer, Waltham, MA, USA) at an excitation wavelength of 490 nm and a detection wavelength of 520 nm. The concentrations were calculated based on standard solutions of FITC‐labeled dextran in PBS medium.

### Statistical analysis

Data are expressed as the mean ± standard deviation (SD) and analyzed using spss version 20.0 (IBM Corp., Armonk, NY, USA). The study was conducted in at least three independent runs and *n* refers to the number of independent experiments. Data were compared by unpaired Student's *t*‐test. *P* < 0.05 was considered statistically significant.

## Results

### Effects of different dosages of escin and fixed dosage of HMGB1 on cell viability in ECs

**Figure 1:**
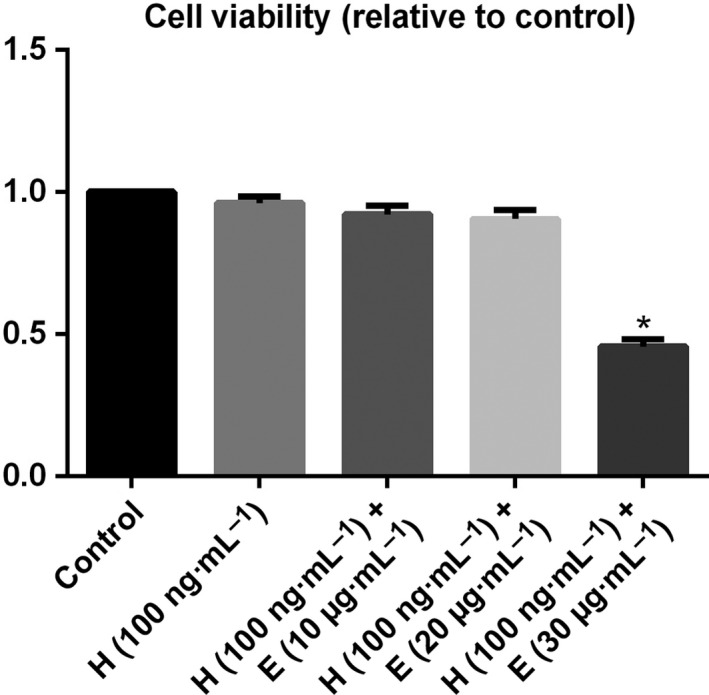
Effects of different dosages of escin and fixed dosage of HMGB1 on cell viability in ECs. ECs were treated with medium only, HMGB1 (100 ng·mL^−1^) alone or escin (10, 20, or 30 μg·mL^−1^) with HMGB1 (100 ng·mL^−1^) for 24 h. Cell viability was assessed using MTT reduction assays. Data from three independent experiments were pooled. Data are presented as mean ± SD, *n* = 3. *P < 0.05, compared with the control by unpaired Student's *t*‐test. H, HMGB1; E, escin.

ECs were treated with an increasing concentration (10, 20, or 30 μg·mL^−1^) of escin in the presence of HMGB1 (100 ng·mL^−1^) or incubated with HMGB1 (100 ng·mL^−1^) alone for 24 h, and the effect of escin on cell survival was detected with an MTT assay. There was no obvious toxic effect on the activity of ECs with the fixed concentration (100 ng·mL^−1^) of HMGB1 and EC activity decreased with increasing concentration of escin, in which 10 or 20 μg·mL^−1^ escin accompanied by HMGB1 (100 ng·mL^−1^) showed some damage to cells, but no statistical significance was found (*P* > 0.05). Escin at 30 μg·mL^−1^ accompanied by HMGB1 (100 ng·mL^−1^) showed obvious damage to the ECs and statistical significance was found (*P* < 0.05). Details are shown in Fig. 1.

### HMGB1 increases the expression of AQP1 and escin reverses the overexpression of AQP1 in ECs

**Figure 2:**
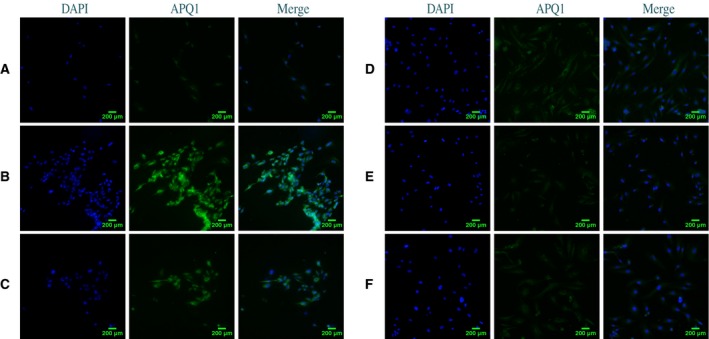
AQP1 immunofluorescence changes after treated with HMGB1 or HMGB1 combined with escin in cultured ECs. (A) Normal AQP1 expression intensity in ECs. (B) Treatment with HMGB1 (100 ng·mL^−1^) for 24 h significantly increased AQP1 expression intensity and produced some deformity of ECs. (C) Treatment with HMGB1 (100 ng·mL^−1^) combined with escin (10 μg·mL^−1^) for 24 h led to a comparatively reduced AQP1 expression intensity. (D–F) AQP1 expression intensity was significantly decreased when ECs were treated with HMGB1 (100 ng·mL^−1^) combined with escin (20 μg·mL^−1^) for 12, 18, or 24 h. Scale bar =  200 μm.

**Figure 3:**
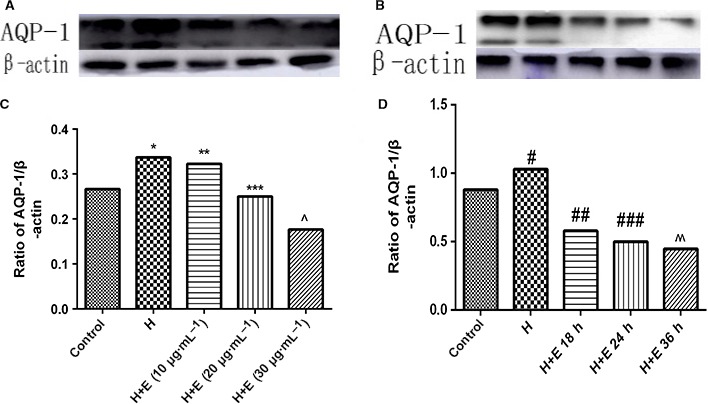
Western blot analysis of expression of AQP‐1 after treated with HMGB1 or HMGB1 combined with escin in cultured ECs. (A) Representative western blot band images for AQP‐1 after challenge with control, 100 ng·mL^−1^ HMGB1, 100 ng·mL^−1^ HMGB1 + 10 μg·mL^−1^ escin, 100 ng·mL^−1^ HMGB1 + 20 μg·mL^−1^ escin or 100 ng·mL^−1^ HMGB1 + 30 μg·mL^−1^ escin at 37 °C for 24 h. (B) Relative ratio of band intensities of AQP‐1 over β‐actin after challenge with control, 100 ng·mL^−1^ HMGB1, 100 ng·mL^−1^ HMGB1 + 10 μg·mL^−1^ escin, 100 ng·mL^−1^ HMGB1 + 20 μg·mL^−1^ escin or 100 ng·mL^−1^ HMGB1 + 30 μg·mL^−1^ escin at 37 °C for 24 h. (C) Representative western blot band images for AQP‐1 after challenge with control, 100 ng·mL^−1^ HMGB1 + 20 μg·mL^−1^ escin for 12, 18, or 24 h, respectively. (D) Relative ratio of band intensities for AQP‐1 over β‐actin after challenge with control, 100 ng·mL^−1^ HMGB1 + 20 μg·mL^−1^ escin for 12, 18, or 24 h, respectively. Band intensities were acquired by image j and presented as mean ± SD, *n* = 3. Data were compared by unpaired Student's *t*‐test. *P < 0.05 *vs* control group; **P < 0.05 *vs* H group; ***P < 0.05 *vs* H + E (10 μg·mL^−1^) group; ^*P* < 0.05 *vs* H + E (20 μg·mL^−1^) group; ^#^ *P* < 0.05 *vs* control group; ^##^ *P* < 0.05 *vs* H group; ^###^ *P* < 0.05 *vs* H + E 18 h group; ^^*P* < 0.05 *vs* H + E 24 h group. H, HMGB1; E, escin.

Immunocytochemistry was used to measure expression intensity of AQP1 in cultured ECs. In the immunocytochemical assay, ECs grown on glass coverslips were treated with a fixed concentration (100 ng·mL^−1^) of HMGB1 or fixed concentration (100 ng·mL^−1^) of HMGB1 and escin (10 μg·mL^−1^) or control for 24 h or fixed concentration (100 ng·mL^−1^) of HMGB1 and fixed concentrations (20 μg·mL^−1^) of escin for 12, 18, 24 h. AQP1 was slightly expressed in the ECs in the control group, but when challenged with recombinant HMGB1, a significant immunofluorescence intensity increase was produced. However, the immunofluorescence intensity was markedly downregulated following treatment with escin in a dose‐ and time‐dependent manner; details are shown in Fig. 2. Interestingly, we found that to some extent there was a favorable effect on the changed shape of ECs caused by HMGB1 with an increase in incubation time and concentration. The protein quantity of AQP1 was determined by western blot assay. In the cultured ECs, a high quantity of AQP1 was observed with HMGB1 administration. Treatment with 100 ng·mL^−1^ HMGB1 increased the expression of AQP1 in cultured ECs, but AQP1 quantity was decreased when HMGB1 was accompanied by escin administration, with increased reduction with the increase of escin dosage and actuation duration. Details are shown in Fig. 3. In agreement with the immunocytochemical assay, a dose‐dependent and time‐dependent effect was seen in a western blot assay.

### HMGB1 induces actin cytoskeletal rearrangement and escin can protect against HMGB1‐induced EC barrier disruption

**Figure 4:**
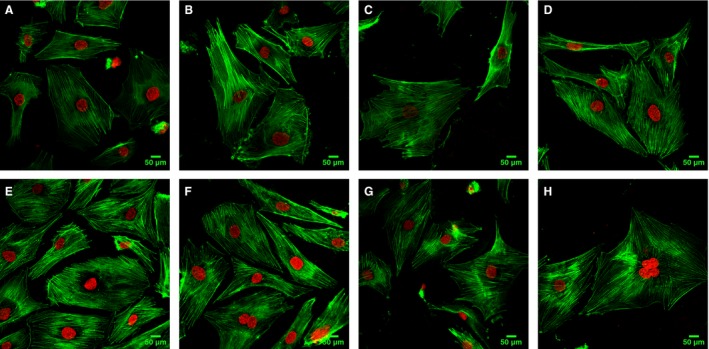
The organization of F‐actin, shown as green fluorescence, was observed using confocal microscopy. Nuclei are shown by DAPI staining (red fluorescence). (A) F‐actin distribution in normal ECs. (B) Treatment with HMGB1 (100 ng·mL^−1^) for 24 h increased thick actin stress fiber formation in the central of ECs. (C) Treatment with HMGB1 (100 ng·mL^−1^) combined with escin (10 μg·mL^−1^) for 24 h resulted in comparatively thick F‐actin fibers evenly distributed in the ECs. (D) F‐actin tended to show close to normal morphology when treated with HMGB1 (100 ng·mL^−1^) combined with escin (20 μg·mL^−1^) for 24 h. (E) Treatment with HMGB1 (100 ng·mL^−1^) combined with escin (30 μg·mL^−1^) for 24 h accelerated gathering of F‐actin in the periphy of the cells. (F) Treatment with HMGB1 (100 ng·mL^−1^) combined with escin (20 μg·mL^−1^) for 12 h resulted in a comparatively clustered arrangement of thick F‐actin in the centre of the cells as compared with (D,H). (G,H) F‐actin was restored to close to normal morphology when treated with HMGB1 (100 ng·mL^−1^) combined with escin (20 μg·mL^−1^) for 18 or 24 h.

ECs on glass coverslips were grown to 70–80% confluency then treated with 100 ng·mL^−1^ HMGB1 or 100 ng·mL^−1^ HMGB1 accompanied with varying concentrations (10–30 μg·mL^−1^) of escin. In this study, prior to HMGB1 and escin stimulation, the majority of F‐actin was evenly distributed in cells (Fig. 4A). When the ECs were exposed to HMGB1 for 24 h, thick actin stress fibers tended to gather in the center of the cell, with loss of peripheral organized actin fibers, a phenotypic change associated with increased contractile activity and increased endothelial permeability as previously described in the literature 17, 18 (Fig. 4B). However, when the cells were exposed to HMGB1 accompanied with escin (10, 20, or 30 μg·mL^−1^) for 24 h, inhibition of F‐actin reorganization was found, as shown in Fig. 4C–E. A decreased gathering of thick actin stress fibers in the center of the cell occurred when cells were treated with 10 μg·mL^−1^ escin (Fig. 4C). Further, endothelial F‐actin was restored to close to normal morphology with 20 μg·mL^−1^ escin treatment (Fig. 4D), with decreased F‐actin thickness compared with HMGB1 administration alone. A tendency of F‐actin to gather in the periphery of the cells was found with 30 μg·mL^−1^ escin treatment (Fig. 4E). Moreover, when the cells were treated with HMGB1(100 ng·mL^−1^) and escin (20 μg·mL^−1^) for 12, 18 and 24 h, a significant inhibition of F‐actin reorganization was seen over time, and the morphology of the endothelial F‐actin with a long incubation time was similar to the normal morphology (Fig. 4F–H). Thus, we can draw the conclusion that escin can protect ECs against HMGB1‐induced cytoskeletal changes with decreased central stress fiber formation in a dose‐ and time‐dependent manner.

### HMGB1 increases endothelial monolayer permeability, which could be reversed by escin

**Figure 5:**
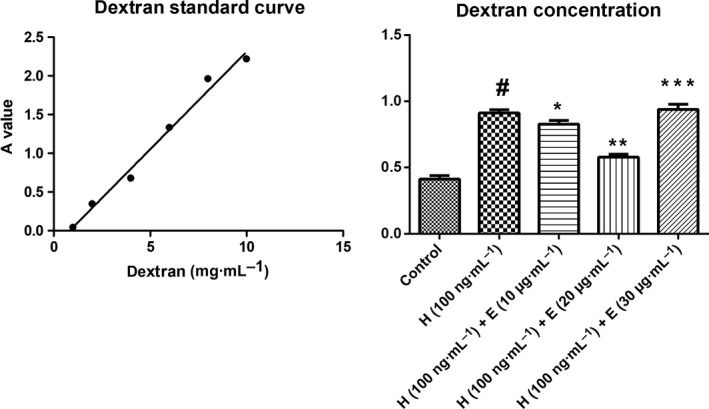
Effects of HMGB1 and escin on FITC‐dextran diffusion through the human endothelial cell monolayer. Cells were challenged with control, 100 ng·mL^−1^ HMGB1, 100 ng·m^−1^ HMGB1 + 10 μg·mL^−1^ escin, 100 ng·mL^−1^ HMGB1 + 20 μg·mL^−1^ escin or 100 ng·mL^−1^ HMGB1 + 30 μg·mL^−1^ escin at 37 °C for 24 h, and the amount of FITC‐dextran diffused through the monolayer was measured 90 min after its addition on top of the monolayer. The amount of FITC‐dextran was normalized according to a standard curve; data from three independent experiments were pooled and presented as mean ± SD. Data were compared by unpaired Student's *t*‐test. #*P* < 0.05 *vs* control group; *P < 0.05 *vs* H group; **P < 0.05 *vs* H (100 ng·mL^−1^) + E (10 μg·mL^−1^) group; ***P < 0.05 *vs* H (100 ng·mL^−1^) + E (20 μg·mL^−1^) group. H, HMGB1; E, escin. Scale bar =  50 μm.

We treated ECs with HMGB1 at 100 ng·mL^−1^ or HMGB1 accompanied with escin (10, 20, or 30 μg·mL^−1^) in culture medium for 24 h. Endothelial monolayers exposed to medium only (controls) demonstrated a slight increase in permeability to FITC‐labeled dextran over time. However, endothelial cell monolayers exposed to HMGB1 showed a significantly greater increase in permeability to FITC‐labeled dextran compared with control monolayers. Interestingly, endothelial cell monolayers exposed to HMGB1 combined with escin showed a significant decrease in permeability to FITC‐labeled dextran; however, we suspected that the increase of permeability to dextran in the 30 μg·mL^−1^ escin group was caused by the toxic effect of escin at high concentration. Figure 5 demonstrates the effect of varying concentrations of escin (10, 20, and 30 μg·mL^−1^) on endothelial cell monolayer permeability. We found that incubation of HMGB1 increases HUVEC permeability to FITC‐labeled dextran and escin may have some curative effect on the process.

## Discussion

Escin, isolated from traditional herbal medicines, is widely used for the treatment of several illnesses in the clinic, ranging from CVI to post‐operative edema 7. However, the anti‐edematous effect of escin on the expression of AQP1 and barrier protective functions in human ECs have not been studied yet. Here, we have identified escin as a natural AQP1 modulator that can inhibit the expression of AQP1 in ECs during HMGB1 stimulation. In the present study, we found that escin prevented HMGB1‐associated hyperexpression of AQP1. Moreover, the inhibition of AQP1 caused by the stimulation of HMGB1 was still significant at toxic concentrations. Also, we found that HMGB1 accelerated the gathering of thick actin stress fibers in the center of the ECs and that increasing concentrations of escin could inhibit this F‐actin reorganization and accelerate F‐actin accumulation at the periphery of the cells. Then we demonstrated that HMGB1 increases endothelial monolayer permeability and this could be reversed by escin. This study is the first to demonstrate the feasibility of utilizing escin to reduce the hyperexpression of AQP1 and hyperpermeability induced by HMGB1, and to provide the anti‐edematous mechanism of escin as well as its protective role at cell barriers in ECs.

Aquaporin 1 is a membrane protein that can control the permeability of endothelial and epithelial barriers by facilitating water movement across cell membranes. Currently, it has been confirmed that AQP1 is associated with multiple different illnesses including abnormalities of kidney function, onset of brain edema and spinal cord injury 19, 20. Of these, reduced water permeability, defective proximal tubular fluid resorption, and severely impaired urinary concentrating ability were found in transgenic mice lacking AQP1 water channels, which is predicted to produce water diuresis by a mechanism that is different from conventional salt transport‐blocking diuretics 21, 22, 23. Thus, AQP1 blockers could have clinical potential in the refractory edema that is associated with congestive heart failure and cirrhosis. Moreover, increased AQP1 expression was seen in injured spinal cords, which is associated with neuronal and astrocytic swelling and the development of neuropathic pain after spinal cord injury 20. Due to its specific role in water exchange, AQP1 may represent a potential therapeutic target in the management of water imbalance disorders and its modulators might decrease the permeability of barrier and serve as useful anti‐edema agents, which could have a curative effect on various type of edema mediated by AQP1. The anti‐edematous effect of escin has already been confirmed in an *in vivo* rat model, where escin attenuated cerebral edema induced by acute omethoate poisoning; the underlying mechanism was probably associated with amelioration of the permeability of the blood–brain barrier 24. However, to date few AQP inhibitors have been discovered as suitable candidates for clinical development. Currently, the sulfhydryl‐reactive, heavy metal‐containing compounds and some compounds isolated from medicinal plant have been confirmed as AQP1 inhibitors; however, there is still a need to identify useful inhibitors with therapeutic application in the clinic and limited side effects 25, 26. In our study, we found a new agent from natural products that can inhibit the expression of AQP1 in a dose‐dependent and time‐dependent manner and improve barrier dysfunction.

Caused by disruption of vascular integrity, an increase in permeability is an essential feature of the inflammatory process in vascular inflammatory diseases, and may contribute significantly to high morbidity and mortality 27. HMGB1, a ubiquitous nuclear protein and important pro‐inflammatory mediator, usually participates in the evolution of infectious and sterile inflammation when released extracellularly after cellular activation, stress, damage or death, and has emerged as a therapeutic target in inflammatory diseases 28. Agents that exhibit the capacity to reverse increases in vascular permeability, a prominent feature in diverse inflammatory syndromes, would inhibit fluid flux into the interstitium and have obvious therapeutic applications. Previous literature has demonstrated that HMGB1 could induce paracellular gap formation as well as barrier dysfunction, resulting in an increase in the permeability of ECs 2. In accordance with current findings, we verified that HMGB1 increased the permeability of ECs, indicating that membrane‐associated AQP1 and barrier dysfunction possibly occurred during the process. Thus, based on the current findings, our study showed that HMGB1 could increase the expression of AQP1 and induce barrier dysfunction, suggesting that HMGB1 might play a role in fluid accumulation and edema via AQP1 and that HMGB1‐induced hyperpermeability might be associated with barrier dysfunction.

The F‐actin cytoskeletal elements are anchored to the intercellular junctional proteins, and reorganization of these elements is associated with loss of cell–cell contact, cell retraction, and intercellular gap formation 29. In our study, HMGB1 could induce formation of central thick actin stress fibers with loss of peripheral organized actin fibers, which possibly results in increased endothelial permeability causing increased interstitial edema. In support of this, changes in F‐actin distribution and loss of the peripheral F‐actin ring are also observed in endothelium in response to other pro‐inflammatory agonists 30. Thus, in our experiment, in response to HMGB1 stimulation, we found F‐actin redistribution associated with intercellular gap formation and formation of transcellular gaps, by the activity of AQP1, might play an important role in the HMGB1‐associated inflammatory response.

The pharmacodynamics of escin, its anti‐edematous properties, anti‐inflammatory activities and venotonic properties, are widely accepted in clinical application. Of these, the anti‐edematous properties have already been proven in models of inflammation, whose mechanism mainly includes sensitization to Ca^2+^ ions, a ‘sealing effect’ on small permeable vessels and an increase of venous tone 7. In our models of inflammation *in vitro*, we found a decreased expression of membrane‐associated AQP1, a molecule correlated with water permeability, in cells treated with escin, and this might be associated with reduced permeability of vessels to water. Moreover, with the increase of escin concentration, decreased thick actin stress fiber formation and a tendency of F‐actin to gather in the periphery of the cells was found, which might be associated with decreased contractile activity and inhibited gap formation. Taken together, combined with our results, we can draw the conclusion that escin is a desirable agents for a variety of inflammatory diseases, which can not only inhibit the inflammatory response but also decrease the expression of AQP1 and enhance EC barrier function in order to reduce inflammation‐associated edema.

We found that HMGB1‐induced formation of actin stress fibers might lead to cellular contraction and disruption of the endothelial barrier, thereby inducing an increased permeability of the endothelial monolayer. Furthermore, we found that formation of transcellular gaps, namely by the activation of AQP1, still plays an important role during this process. Nevertheless, which of them plays a dominant role still needs to be elucidated. However, this effect was attenuated by exposing ECs to escin. The protective effects of escin may result from inhibition of AQP1 and stabilization of cytoskeletal fibers or even enhancement of the peripheral F‐actin rim that counteracts the barrier‐disruptive effects of HMGB1. Our observations showed that formation of both transcellular gaps and intercellular gaps plays a vital role in the HMGB1‐induced inflammatory response and escin could reverse this kind of pathological change, resulting in inhibition of biological membrane permeability to water and consequently reduction of the fluid content of body tissues and organs. We concluded that escin enhanced the stability and integrity of the endothelial barrier together with a decrease in the expression of AQP1, suggesting it has potential as a therapeutic agent against edema as well as pro‐inflammatory diseases.

In conclusion, our findings indicated that escin suppressed HMGB1‐induced AQP1 hyperexpression of ECs even at toxic concentration. Furthermore, HMGB1 could still increase the permeability of ECs and this kind of pathological change could be reversed by escin, suggesting that escin has a curative effect on HMGB1‐induced barrier dysfunction via inhibiting AQP1 and protecting the integrity of the endothelial barrier. Thus we can conclude that escin is a desirable agent in dealing with inflammatory hyperpermeability and even edema and could be widely used in the clinic; however, future studies are needed to obtain the active ingredients of escin with lower toxicity and more efficiency 31.

## Conflict of interest

The authors declare no conflict of interest.

## Author contributions

CC and MS conceived and designed the project. CC, SW, JC, XL, MZ, WX and XW acquired the data. XP and MS analyzed and interpreted the data. YZ, HL, XP and CC wrote the paper.
